# Publication trend, resource utilization, and impact of the US National Cancer Database: A systematic review

**DOI:** 10.1097/MD.0000000000009823

**Published:** 2018-03-02

**Authors:** Chang Su, Cuiying Peng, Ena Agbodza, Harrison X. Bai, Yuqian Huang, Giorgos Karakousis, Paul J. Zhang, Zishu Zhang

**Affiliations:** a Department of Radiology, The Second Xiangya Hospital, Central South University, Changsha, Hunan, China; b Yale University School of Medicine, New Haven, CT, USA; c Department of Neurology, The Second Xiangya Hospital, Central South University, Changsha, Hunan, China; d University of Pennsylvania; e Department of Radiology, Hospital of the University of Pennsylvania; f Department of Surgery, Hospital of the University of Pennsylvania; g Department of Pathology and Laboratory Medicine, Hospital of the University of Pennsylvania, Philadelphia, PA, USA.

**Keywords:** bibliometrics, National Cancer Database, publication trend, resource utilization

## Abstract

**Background:**

The utilization and impact of the studies published using the National Cancer Database (NCDB) is currently unclear. In this study, we aim to characterize the published studies, and identify relatively unexplored areas for future investigations.

**Methods:**

A literature search was performed using PubMed in January 2017 to identify all papers published using NCDB data. Characteristics of the publications were extracted. Citation frequencies were obtained through the Web of Science.

**Results:**

Three hundred 2 articles written by 230 first authors met the inclusion criteria. The number of publications grew exponentially since 2013, with 108 articles published in 2016. Articles were published in 86 journals. The majority of the published papers focused on digestive system cancer, while bone and joints, eye and orbit, myeloma, mesothelioma, and Kaposi Sarcoma were never studied. Thirteen institutions in the United States were associated with more than 5 publications. The papers have been cited for a total of 9858 times since the publication of the first paper in 1992. Frequently appearing keywords congregated into 3 clusters: “demographics,” “treatments and survival,” and “statistical analysis method.” Even though the main focuses of the articles captured a extremely wide range, they can be classified into 2 main categories: survival analysis and characterization. Other focuses include database(s) analysis and/or comparison, and hospital reporting.

**Conclusion:**

The surging interest in the use of NCDB is accompanied by unequal utilization of resources by individuals and institutions. Certain areas were relatively understudied and should be further explored.

## 1 Introduction

The National Cancer Database (NCDB) is a community-oriented cancer management and outcomes database that is a joint project of the Commission on Cancer of the American College of Surgeons and the American Cancer Society.^[1]^ More than 1500 Commission on Cancer (CoC)-accredited facilities contribute to the NCDB, and the database encompasses data on patient demographics, tumor characteristics, socioeconomic factors, treatments, and survival outcomes. The NCDB dataset contains more than 34 million historical records, representing more than 70% of all newly diagnosed cancer cases nationwide.^[2]^ Furthermore, as a data registry that gives feedback to participating institutions, NCDB not only supports cancer research endeavors, but also helps to improve the quality of cancer care in the United States.^[3]^

Bibliometrics is an important statistical tool that analyzes written publications, including books and academic papers, to measure scientific output of an individual, an institution, and/or a country, using relevant parameters including quantity, impact factor, and citations of the published articles over time.^[4]^ In bibliometric studies, the characteristics and metrology of a system of literature serve as the research object, and the literature are analyzed both quantitatively and qualitatively.^[5]^ In particular, bibliometrics can be used to characterize the current status of research using a certain data registry, which can help to monitor its growth and utility pattern by researchers.^[6]^ Furthermore, bibliometric analysis provides information and a platform for research groups to form collaborations around the world. In the recent years, a growing number of bibliometric studies are published in high impact journals in the field of medicine,^[7–13]^ and journals are transitioning from publishing only conventional research studies to including bibliometric studies.^[14]^

Despite of the surging interest and usage of NCDB in the past few years, the trend of publications in the medical field using data from the NCDB and its impact are currently unclear. Studying the representations of different types of cancers, the institutions associated with the publications, and the focuses and impact of the studies published, among other trends, can help the scientific community determine future research directions using data from the NCDB. In this study, we aimed to characterize the studies using NCDB data that have been published, and to identify relatively unexplored areas that can form the basis for future investigations.

## 2 Method

### 2.1 Sources of the data and search strategy

A literature search was performed using PubMed in January 2017 to identify all papers published in the literature using data from the NCDB. All entries in PubMed were exported into EndNote X7 (Clarivate Analytics, Philadelphia, PA), a reference management software. The full papers in the Portable Document Format were downloaded through EndNote whenever possible, and the papers that were not found via EndNote were downloaded manually. The search keyword was “NCDB” or “National Cancer Database” Ethical approval was not necessary since the data were downloaded from a public databases. Authors did not have access to information that could identify individual participants during or after data collection.

### 2.2 Data collection

The data entry and collection were performed by 2 authors (CP and EA), and any discrepancies were resolved in a panel involving an additional author (HXB). The characteristics of the relevant publications, including country of origin, first institution listed, first author, journal in which the paper was published in, year of publication, cancer categories according to the NCDB, type of publication, and main focuses of the paper, were extracted. Citation frequencies were obtained through the Web of Science. The impact factor of each journal was extracted from the official website of the journal.

### 2.3 Statistical methods

The manually extracted information of the publications was recorded in Microsoft Excel 2016 (Microsoft, Redmond, WA) and analyzed using SPSS V22.0 (SPSS, Inc., Chicago, IL). Figures were made in GraphPad Prism 5 (GraphPad Software, Inc., San Diego, CA) and the Microsoft Office Suit 2016 (Microsoft, Redmond, WA). VOSviewer (Leiden University, Leiden, Netherlands), a commonly used method for cocitation network analysis and visualization,^[15]^ was used to analyze all papers to visualize the relationships among the most frequently occurring concepts and keywords. In addition, knowledge maps of frequencies of the most commonly occurring keywords, clusters of key concepts, and the temporality of keywords were generated in VOSviewer.

## 3 Results

### 3.1 The number of publications

**Figure 1:**
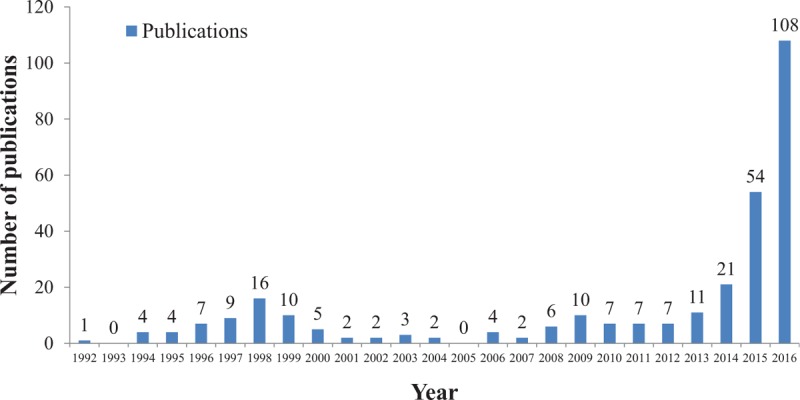
The number of publications involving data from NCDB by year.

A total of 302 articles met the inclusion criteria. As shown in Fig. 1, the number of publications grew exponentially since 2013. The number of papers published since 2015 (2015 and 2016) was more than that of all previous years combined (1992–2014), with a surging number of 108 publications in 2016 alone. Noticeably, the number of publications also peaked around 1998, though the magnitude of the increase was negligible compared to the number of publications in 2015 and 2016.

### 3.2 Cancer categories

The number and proportion of papers that focused on each of the broad cancer categories according to the NCDB is listed in Table 1. The analysis showed that the majority of the publications using data from the NCDB were related to digestive system cancer, while no publication was related to the bone and joints cancers, eye and orbit cancers, myeloma, mesothelioma, and Kaposi Sarcoma.

**Table 1:**
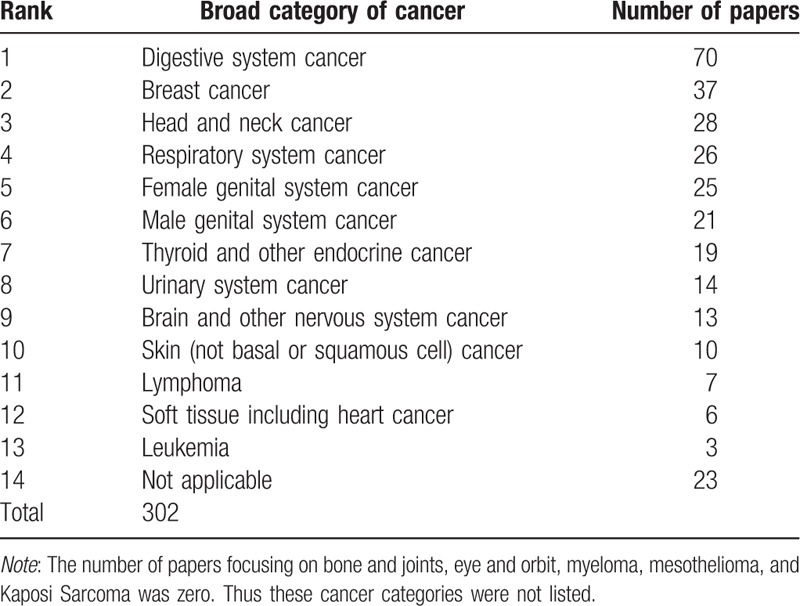
The publication number of broad category of cancer during 1992–2016.

### 3.3 Institutions

The 302 publications come from 134 different institutions. Thirteen institutions were associated with more than 5 publications using data from the NCDB (Table 2). The institution with the most publications was Yale University School of Medicine (n = 20), followed by Duke University Medical Center (n = 13), and the University of Colorado School of Medicine (n = 13).

**Table 2:**
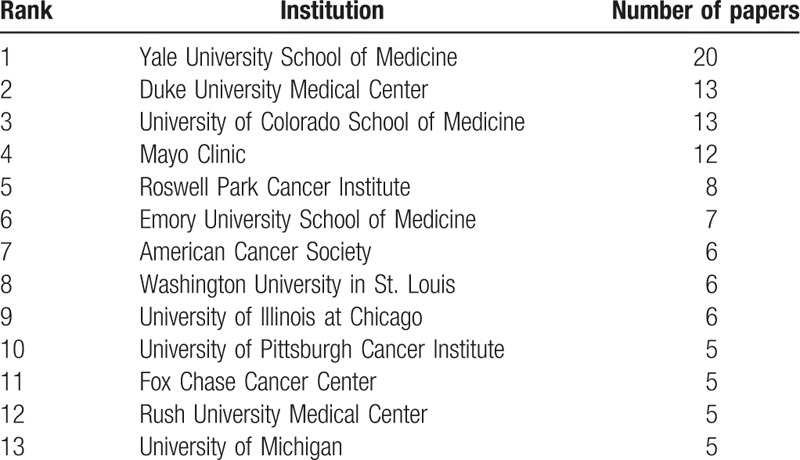
Institutions associated with more than 5 publications using data from the NCDB.

### 3.4 Citation frequency

All papers using data from the NCDB have been cited for a total of 9858 times since the publication of the first paper in 1992. The mean citation frequency for one paper was 32.6. The paper titled “A National Cancer Data Base Report on 53,856 Cases of Thyroid Carcinoma Treated in the U.S., 1985–1995” published in *Cancer* has been cited the highest number of times (n = 1017). The 10 most cited papers using data from the NCDB are shown in Table 3.

**Table 3:**
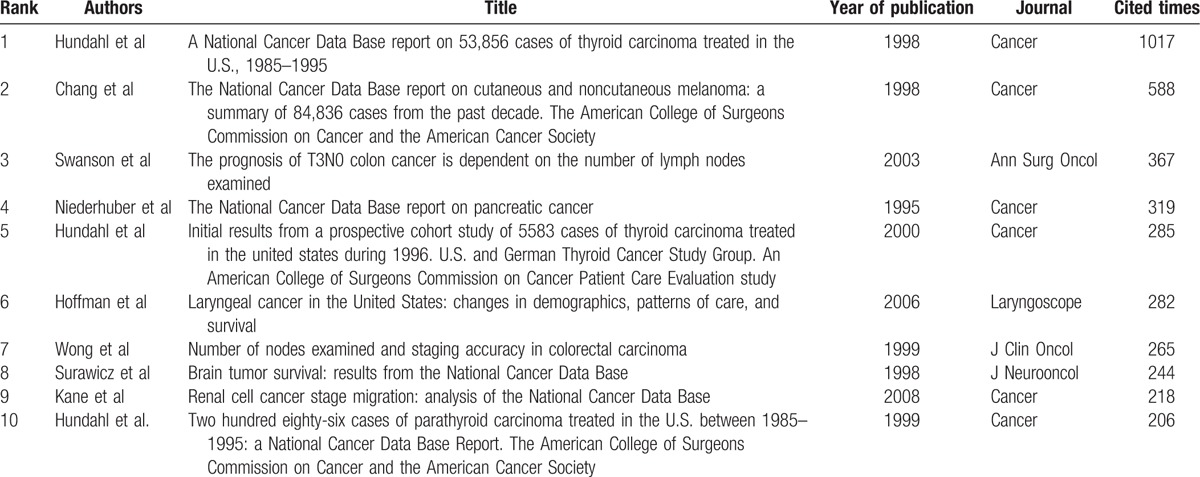
Top 10 most cited articles using data from the NCDB.

### 3.5 Journals

**Figure 2:**
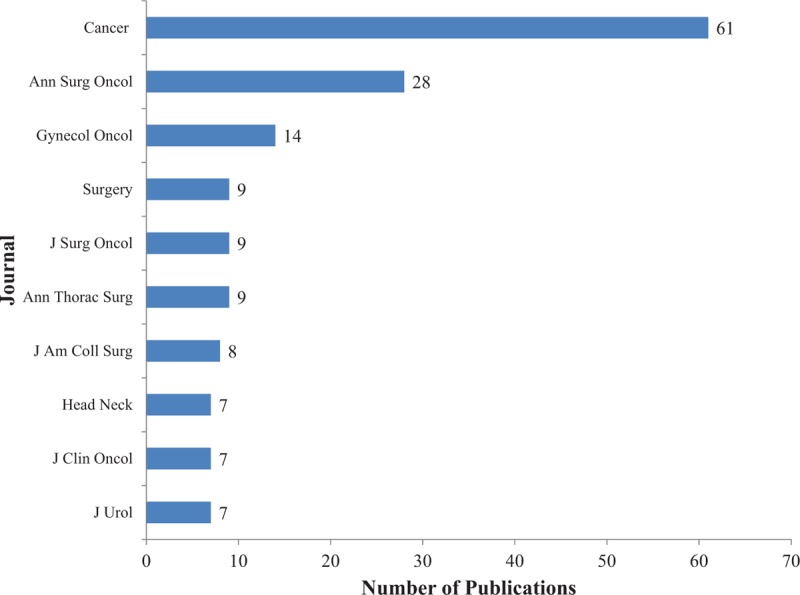
The top 10 journals that published the most papers using data from the NCDB.

Over 86 journals published papers using data from the NCDB. The 10 journals that published the largest number of papers are shown in Fig. 2. The journal *Cancer* published the largest number of papers (n = 61). The 5 journals with the highest impact factors (IF) that published papers using data from the NCDB are shown in Table 4. The journal *CA: A Cancer Journal for Clinicians* is the journal with the highest IF (IF = 137.58) that published papers using data from the NCDB.

**Table 4:**
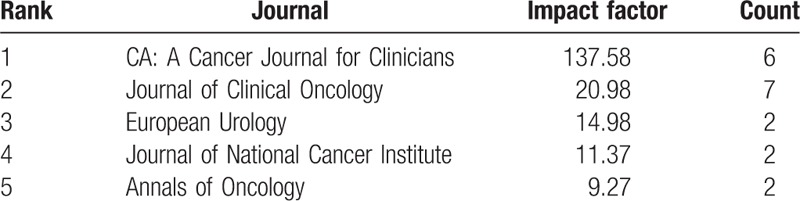
The 5 journals with the highest impact factor that published papers using data from the NCDB.

### 3.6 Authors

Over 230 first authors contributed a total of 302 papers using data from the NCDB. Arya Amini published the highest number of papers (n = 8) using data from the NCDB, followed by Karl Y. Bilimoria (n = 6) and John R. Bergquist (n = 5). Almost all publications by Arya Amini were in 2016, and the author mainly focused on the treatment and survival of intermediate-risk prostate cancer and oropharynx carcinoma. Karl Y. Bilimoria's publications were between 2007 and 2009, and the author mainly focused on melanoma, thyroid cancer, and adrenocortical carcinoma. John R. Bergquist's publications were all published in 2016, and the author focused exclusively on digestive system cancer, mostly on cholangiocarcionoma.

### 3.7 Hotspots

**Figure 3:**
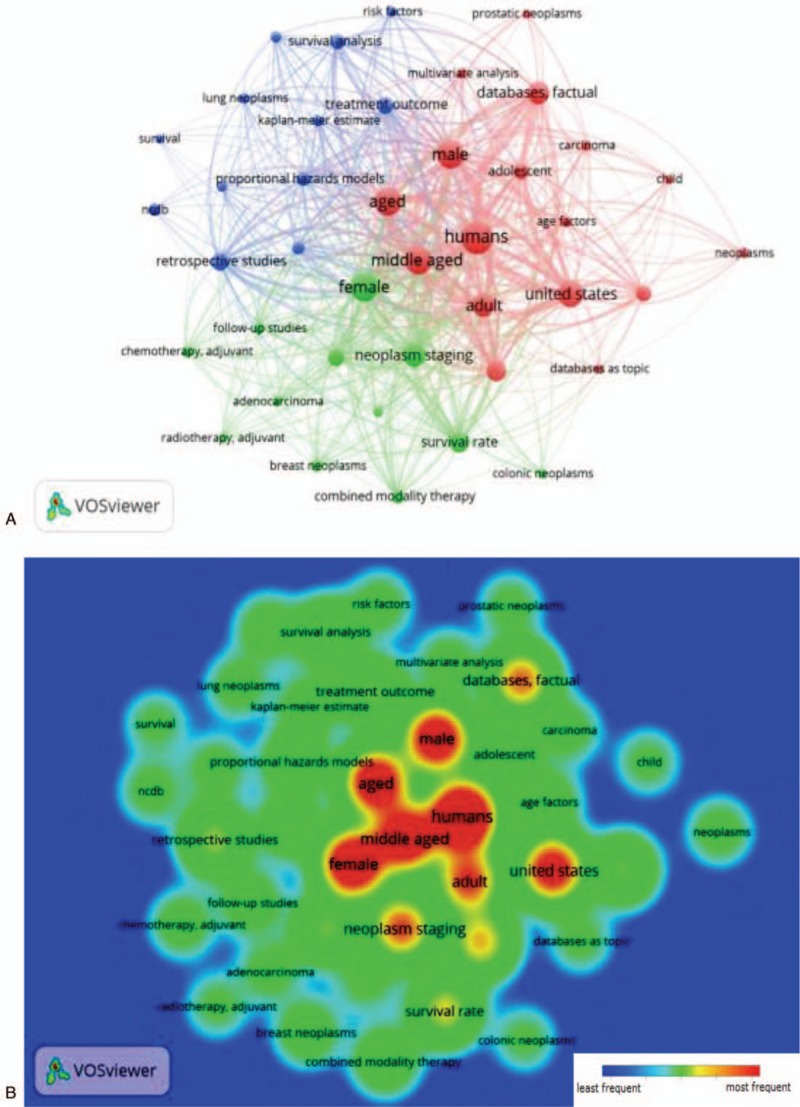
(A) The network visualization of the keywords divided based on clusters from all 302 publications. The “demographics” cluster was red, the “treatments and survival” cluster was green, and the “statistical analysis method” cluster was blue. (B) The density visualization of the frequency of the keywords divided based on clusters from all 302 publications. Red represent the most frequently used words, and blue represent the lest frequently used words.

Keywords of all 302 papers were analyzed using VOSviewer. As shown in Fig. 3, the keywords congregated into 3 clusters: the “demographics” cluster, the “treatments and survival” cluster, and the “statistical analysis method” cluster. Among the “demographics” cluster, keywords, defined as appearing more than 20 times within an article, included human (n = 222), middle age (n = 158), male (n = 154), aged (n = 153), United States (n = 136), adult (n = 108), factual database (n = 96), age 80 and over (n = 79), registries (n = 48), adolescent (n = 42), and age factor (n = 24). For the “treatment and survival” cluster, the keywords included female (n = 172), neoplasm staging (n = 104), survival rate (n = 70), prognostic (n = 50), combined modality therapy (n = 32), breast neoplasm (n = 25), follow-up studies (n = 24), and adjuvant chemotherapy (n = 21). In the “statistical analysis method” cluster, keywords included retrospective studies (n = 60), treatment outcome (n = 51), survival analysis (n = 42), proportional hazards models (n = 34), young adult (n = 31), Kaplan–Meier estimate (n = 23), and time factors (n = 23).

**Figure 4:**
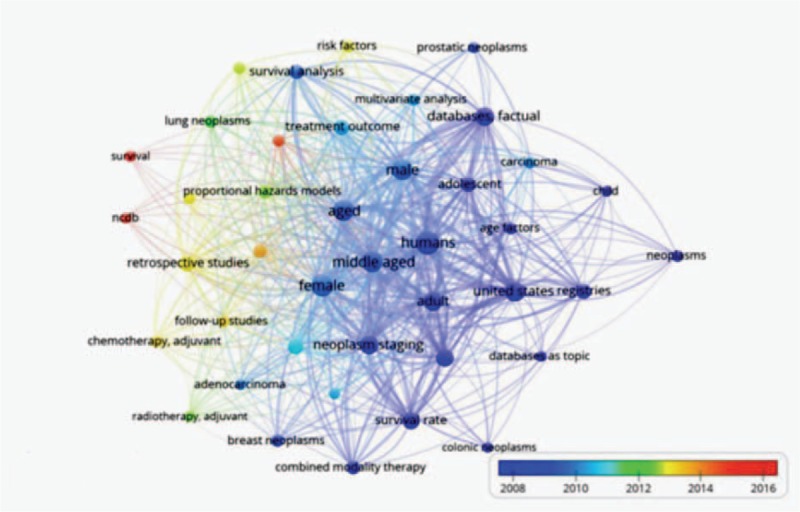
Distribution of keywords according to the time when they first appeared in publications.

A knowledge map showing the distribution of keywords based on the time they appeared in the literature is shown in Fig. 4. As indicated by the color spectrum at the bottom of the figure, the color blue represents keywords that appeared in the earliest publication, while the color red represents keywords that appeared in the most recent publications. In the early stage of research using data from the NCDB, demographics was the main hotspot. However, in recent years, keywords were shifting towards “young adult” (n = 31), “chemotherapy adjuvant” (n = 21), “follow-up studies” (n = 24), and “Kaplan–Meier estimate” (n = 23) in 2013, and “survival” (n = 15), and “NCDB" (n = 18) in 2015–2016.

### 3.8 Main focus

The main focus of the articles captured an extremely wide range. However, the most frequently occurring themes were survival and characterization. Among the 302 papers, 92 papers focused on survival analyses alone, 83 described the clinical and socioeconomic characteristics alone, and 108 papers focused on both survival and characterization. For the remaining 12 papers, 11 focused on database description and comparison, and 1 focused on hospital reporting. Among the 200 papers that focused on survival analyses, 51, 7, and 129 papers found clinical factors alone, socioeconomic factors/patient characteristics alone, and both, respectively, to affect survival significantly, while 13 papers found no significant prognostic factor. The factors that were most commonly found to affect survival significantly included age (n = 103), treatment (n = 100), stage (n = 73), gender (n = 48), and grade (n = 40). The frequency of the most recurrently studied concepts in publications using data from the NCDB is shown in Table 5.

**Table 5:**
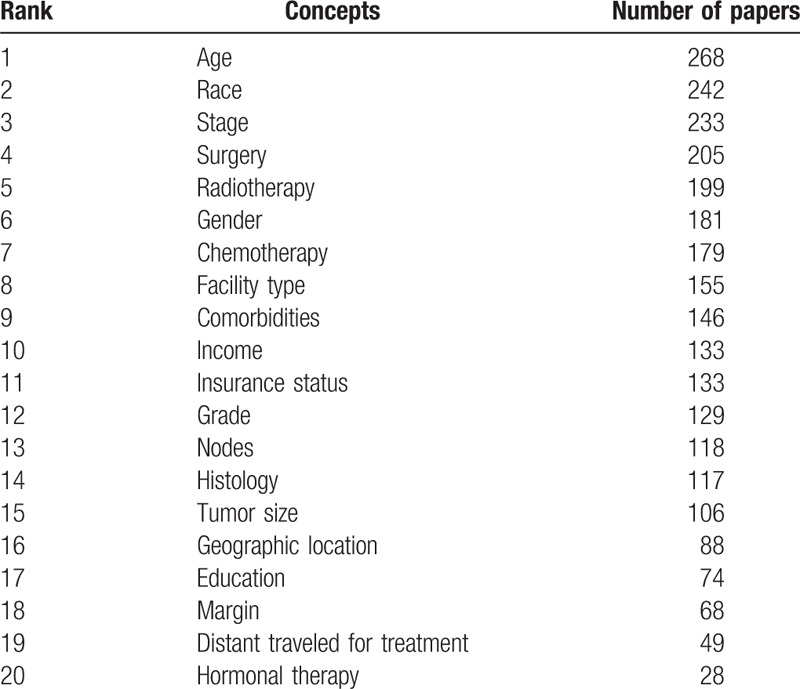
The frequency of the most frequently occurring concepts.

## 4 Discussion and conclusion

Few bibliometric studies have been performed on large databases. In this study, we analyzed the publications that used data from the NCDB to describe overall trend, resource utilization, and impact of the database in the medical field.

Scientific publications are an objective measure of research productivity. Our findings suggest a growing interest in the NCDB database, as demonstrated by the constant increase in the number of publications since 2012. Specifically, a surging number of papers that analyzed data from NCDB were published in the past 2 years (2015 and 2016), suggesting maturity of the database, increased awareness of researchers across the United States to utilize this resource, or more likely, the a combination of both.

From our analysis, the surging interest in using NCDB data can be attributed to a few institutions and authors. Although publications using NCDB data came from 134 different institutions, the top 5 most published institutions accounted for more than 20% of the publications. Among the 230 authors who published papers using NCDB data, the 5 authors (2.1%) who published the highest number of papers were responsible for 29 (9.6%) publications. The publication trend shows an unequal distribution and utilization of the NCDB as a resource for scientific research resulting in publications. Notably, the author who published the most, Arya Amini, published 7 out of his or her 8 papers in 2016. Many authors had more than 1 publication using NCDB data in a single year, mostly in 2016 but some in other years. Such a trend implies that if more investigators have access to the NCDB data, the number of scientific publications will increase greatly in a short period of time. Furthermore, oncology enjoys a disproportionate representation in the most prestigious medical journals.^[16]^ Thus, it is not surprising that nearly 20% of NCDB paper were published in journals with impact IF > 10, with 6 papers published in CA: A Cancer Journal for Clinicians (IF = 137.6).

In addition to the unequal utilization of NCDB data by scientists and institutions, the types of cancers studied using data based on the NCDB was also unequal. Breast, intestinal, and lung cancers, the types of cancers with the highest incidence rate in the United States,^[17]^ were among the most represented types of cancers in publications involving NCDB data. The least studied types of cancers using the NCDB data were leukemia, cancer of the soft tissues including the heart, and lymphoma. In addition, no investigators have studied bone and joint cancer, eye and orbit cancer, myeloma, and mesothelioma using data from the NCDB. Even though soft tissue cancer and mesothelioma are relatively rare, leukemia, lymphoma, central nervous system cancer, and myeloma are among the most represented types of cancers in the medical literature.^[16,18–20]^ Because these types of cancers are of great interest in the scientific community yet not well studied using data from the NCDB, we believe that the NCDB data on these types of cancers should be further explored. Even for rare cancers such as soft tissue cancer and mesothelioma, researchers should take advantage of the large sample size of the NCDB data and further explore these less well-studied types of cancer.^[21,22]^

Besides the unequal representations of different types of cancers, the main focus of the publications is also unequally represented. The majority of publications using NCDB data mainly focused on survival analysis (n = 200) and characterization (n = 191), with 108 papers focusing both on survival and characterization. Only 12 papers focused on themes other than survival or characterization, with 11 focusing on database(s) analysis and/or comparison, and 1 on hospital reporting. The NCDB contains data on a wide range of socioeconomic factors such as facility type, facility location, insurance status, income, education, and the distance travelled for the patient to seek care. We encourage researchers to take advantage of the large and comprehensive data from the NCDB to investigate further into other areas of critically important yet less well-studied fields of clinical research, such as the quality of care and health economics.

We investigated the main focus of the publications through keywords analysis using VOSviewer. The publications using NCDB data were classified into 3 clusters: demographics, treatment and survival, and statistical analysis method. In the demographics cluster, gender and age were the only demographic factors that showed up on the map as the most frequently occurring keywords. In the statistical analysis method cluster, the most frequently appeared keywords included proportional hazards models, Kaplan–Meier estimate, and survival analysis, meaning that the NCDB data were mainly used to analyze the time course of disease progression and factors affecting survival. However, the distribution of keywords according to the time when they first appeared in the literature shows that the research interest has shifted to incorporate more factors over time. Researchers should take advantage of the large sample size and the comprehensiveness of NCDB data, and attempt to analyze data from nontraditional perspectives. For example, the 30-day mortality rate data, which is an indirect measurement of surgical success versus complications, was rarely incorporated in the analysis. In addition, the socioeconomic data, such as income, race, gender, insurance status and education, combined with data on treatment, survival and other disease characteristics, can be used to study disparities in health outcome and healthcare deliveries.^[23–26]^

Although our study provides new knowledge and insights on the present study status in the field of oncology using the NCDB database, we acknowledge a few limitations. First, the results of bibliometric analysis changes with time. Second, since the number of times being cited usually increases with time, the earlier publications potentially show an artificially higher impact than the more recent publications. Third, we searched articles only on PubMed. However, preliminary attempts to use other databases such as Scopus and Embase added few if any additional papers to what was found using PubMed.

In conclusion, the surging interest in the use of NCDB was accompanied by unequal utilization of resources by individuals and institutions. Certain types of cancers, including soft tissue cancer, mesothelioma, leukemia, lymphoma, central nervous system cancer and myeloma, should be further studied.
